# Interventions for COVID-19 infected antenatal patients using symptom severity of symptom

**DOI:** 10.6026/973206300212684

**Published:** 2025-08-31

**Authors:** Somlina Roy, Rubina Dohare, Alluri Rajyalaxmi, Sachin Parmar

**Affiliations:** 1 Department of Obstetrics and Gynecology Amaltas Institute of Medical Sciences, Dewas, Madhya Pradesh, India; 2 Department of Obstetrics and Gynecology Government Medical College, Datia, Madhya Pradesh, India; 3 Department of Community Medicine V.K.S. Government Medical College, Neemuch, Madhya Pradesh, India

**Keywords:** COVID-19 second wave, maternal outcome, neonatal outcome

## Abstract

SARS-CoV-2 infection among pregnant females is of concern. Therefore, it is of interest to evaluate the demographic characteristics, clinical profiles and outcomes of pregnant women with COVID-19 during two pandemic waves. An ambi-directional observational study was conducted at Gandhi Medical College, Bhopal, over 18 months (Jan 2021-Jun 2022), including 210 COVID-19-positive pregnant women. Data showed that the majority were young, unbooked, ruraland illiterate and from lower socioeconomic backgrounds, with anemia being the most common co-morbidity. The second wave saw increased disease severity, ICU interventions and worse maternal and fetal outcomes, including higher maternal mortality (17.3% vs. 4.4%) and stillbirths (11.5% vs. 6.6%). Timely care, early diagnosis and ICU management are critical to reducing morbidity and mortality.

## Background:

Coronavirus disease 2019 (COVID-19), caused by the novel severe acute respiratory syndrome coronavirus 2 (SARS-CoV-2), emerged in Wuhan, China, in December 2019 and rapidly escalated into a global pandemic, resulting in over 5.3 million deaths worldwide. Pregnant women and their offspring have been identified as a particularly vulnerable group during this health crisis. Due to physiological, anatomical and immunological changes, pregnant women face increased susceptibility and potentially more severe outcomes when exposed to infectious diseases, including newly emerging viral pathogens like SARS-CoV-2 [1]. Previous outbreaks of coronaviruses, such as MERS-CoV and SARS-CoV, had maternal mortality rates of 10% and 37% respectively [2]. SARS-CoV-2 shares genetic similarities with these viruses and is transmitted primarily through respiratory droplets and close contacts [3]. High-risk groups include pregnant women, the elderly and individuals with comorbidities [4]. SARS-CoV-2 infection can cause severe disease among pregnant persons [5]. Pregnancy involves complex immunological shifts that may compromise adaptive immunity, thereby reducing the body's ability to clear viral infections efficiently. Anatomical changes, such as an expanded thoracic cage and elevated diaphragm, along with anemia, can diminish respiratory function and oxygen delivery, making expectant mothers less tolerant to hypoxia [6]. Meanwhile, the fetus and neonate, due to their underdeveloped immune systems, are also highly susceptible to infections. Some evidence suggests an enhanced innate immune response may help protect both the mother and fetus [6, 7]. However, current data on vertical transmission of COVID-19 from mother to fetus remain inconclusive, with few confirmed cases and insufficient evidence to establish definitive transmission pathways [8]. Clinical studies have reported adverse maternal and neonatal outcomes during the initial waves of the pandemic. These include an increased risk of spontaneous abortion, preterm premature rupture of membranes (PPROM), preterm birth (PTB), preeclampsia, cesarean delivery, fetal growth restriction (FGR), intrauterine death (IUD) and disseminated intravascular coagulation (DIC). For neonates, complications such as intubation and admission to neonatal intensive care units (NICUs) were also observed [9, 10]. The incidence of preterm birth has been noted to range from 12% to 47%, with symptomatic mothers having a threefold higher rate compared to asymptomatic ones. Some pregnancies required early termination to improve maternal outcomes due to severe disease progression and associated complications [10]. The pandemic also led to widespread disruptions in routine antenatal care (ANC). Health systems adapted by reducing the number of in-person visits, limiting diagnostic procedures to urgent cases and promoting telemedicine services. These modifications significantly impacted the management of pregnancy and have likely influenced maternal and neonatal morbidity and mortality patterns [11]. Therefore, it is of interest to evaluate demographic characteristics, clinical profiles, and outcomes of pregnant women with COVID-19 during two pandemic waves highlight several critical aspects.

## Materials and Methods:

This study was conducted in the Department of Obstetrics and Gynaecology at Gandhi Medical College, Bhopal.

## Study design:

A prospective and retrospective (ambi-directional) observational study.

## Study site:

The study was carried out at the Department of Obstetrics and Gynaecology, Sultania Zanana Hospital & Gandhi Medical College, Bhopal.

## Study duration:

The study was conducted over a period of 18 months, from 1st January 2021 to 30th June 2022.

## Study population:

All pregnant women who tested positive for COVID-19 and reported to the Department of Obstetrics and Gynaecology at Gandhi Medical College, Bhopal, during the study period.

## Sample size:

The study included all COVID-19 positive pregnant women who delivered in Sultania Hospital or the dedicated COVID block at Gandhi Medical College, Bhopal, during the data collection period.

## Inclusion criteria:

[1] Pregnant women who tested positive for COVID-19.

[2] Patients who were willing to provide informed consent.

## Exclusion criteria:

[1] Pregnant women who tested negative for COVID-19.

[2] Women who were not willing to provide informed consent.

## Method of data collection:

After obtaining ethics committee approval (Certificate No: 80/1IEC/2021) and informed consent, data were collected using a prestructured proforma. COVID-19 testing followed ICMR guidelines, with swabs sent in VTM. Symptomatic and asymptomatic cases were managed at Hamidia COVID care center. Baseline demographics, medical history, risk factors, investigations (hemogram, viral markers, USG, HRCT) and antenatal/intranatal details were recorded. Maternal outcomes (e.g., ICU admission, PROM, mortality) and fetal outcomes (e.g., NICU admission, birth weight, anomaliesand COVID-19 status) were assessed per standard protocols.

## Data analysis:

Data was collected and entered into the Statistical Package for Social Sciences (SPSS) version 23. Descriptive statistics were used to summarize the sample characteristics in terms of frequency and percentage and graphical representations were made. Analytical and inferential analyses were performed, with statistical significance set at a p-value of <0.05.

## Consent:

Written informed consent was obtained from all participants. The purpose of the study was thoroughly explained in a language the participants could understand and they were assured of their right to withdraw from the study at any time. Confidentiality of patient information was strictly maintained. The majority of pregnant women were young (18-25 years), unbooked, rural residents, illiterate and from lower socioeconomic backgrounds. Half were in the first trimester and most were multigravida. Anemia was the most common co-morbidity. Fever and breathlessness were predominant symptoms, with severity markedly higher in the second wave. Asymptomatic cases significantly declined. The second wave showed higher ICU needs and more invasive support. Vaginal delivery remained most common. Stillbirths, NICU admissions and maternal deaths increased.

## Results:

The majority of the 210 participants were aged 18-25 years (50.9%), unbooked (67.6%), from rural areas (52.8%), illiterate (46.3%), and belonged to the lower socio-economic class (51.9%). Half of them presented in the first trimester, and most of them were multigravida (63.8%) (Table 1 - see PDF). Anemia (47.7%) was the most common medical comorbidity, and hypertensive disorders of pregnancy (13%) were the most common obstetric complication. Fever was the most common symptom, going from 60.0% in the first wave to 72.5% in the second. Breathlessness went from 18.8% to 46.6%, and cases with no symptoms went from 10% to 0.8%. In the first wave, mild disease was more common (64.4%), but it dropped to 28.7% in the second wave. In contrast, severe cases rose from 4.7% to 17.3% (Table 2 - see PDF). The main ICU treatment in both waves was oxygen through a face mask (30.5% vs. 49%). In the second wave, mechanical ventilation was needed more often (17.5% vs. 4.4%). Vaginal delivery continued to be the predominant mode of delivery (58.8% compared to 57.5%); however, adverse outcomes escalated, with term live births decreasing from 64.4% to 53.7%, stillbirths/intrauterine fetal deaths increasing from 6.6% to 11.5%, NICU admissions rising marginally, and maternal mortality nearly quadrupling from 4.4% in the first wave to 17.3% in the second (Table 3 - see PDF).

## Discussion:

This hospital-based observational study evaluated maternal and fetal outcomes among pregnant women with COVID-19 at Hamidia Hospital, Bhopal. Of the 210 participants, 90 were admitted during the first wave and 120 during the second. Similar to Malik *et al.* [9], the majority of patients in the first wave outnumbered those in the second. Agrawal *et al.* [12] also observed increased admissions during festival seasons and surges. Most participants were aged 18-25 years (50%), reflecting early marriage trends in India, with a majority being unbooked (67.6%), rural (52.8%), illiterate (46.3%), from the lower-middle class and multigravida (63.8%), consistent with previous studies by Chavan *et al.* [13] and Agrawal *et al.* [14]. Anaemia (47.7%) and hypothyroidism (6.8%) were the most common comorbidities, as reported in similar proportions by the Preg Covid registry [10]. Obstetric complications like HDP (13%) and PROM (8%) were common. Similar comorbidity patterns were reported by Parihar *et al.* [11] and Khan *et al.* [15]. COVID symptomatology varied by wave: 64.4% had mild symptoms in the first wave, while 71.3% had moderate to severe symptoms in the second. Oxygen support was needed in 79.5% of cases and 21.9% required mechanical ventilation-higher during the second wave. Neha *et al.* [12] and Sentilhes *et al.* [16] also reported increased oxygen and ventilator requirements. Vaginal delivery was the most common mode (58.8% and 57.5%), followed by LSCS, with fetal distress as the leading indication (56.3%). These trends align with Martínez-Pérez *et al.* [17] and Parihar *et al.* [10]. Increased C-sections were attributed to fetal compromise, not directly to COVID-19 infection. In terms of fetal outcomes, term live births were most frequent (64.4% and 53.7%), but NICU admissions and respiratory distress rates were higher during the second wave. IUFD rates also rose (6.6% to 11.5%). These findings correlate with Basu *et al.* [18], Chavan *et al.* [13] Steven *et al.* [19] and Nayak *et al.* [20], who reported higher preterm births and NICU admissions in COVID-positive pregnancies. Vertical transmission was low, with no conclusive evidence. Nayak *et al.* [20] and Kumari *et al.* [21] reported low neonatal positivity. Gajbhiye *et al.* [22] found a transmission rate of 11%, though generally, vertical transmission remains rare. Maternal mortality was higher during the second wave (17.3%) versus the first (4.4%). Similar to Agrawal *et al.* [13], most patients recovered well. Severe disease, comorbidities and need for ventilation were key mortality drivers, consistent with findings by Di Mascio *et al.* [23].

## Conclusion:

During the COVID-19 pandemic, pregnant women suffered significantly. Poor maternal and fetal outcomes were caused by inadequate prenatal care and increased infection severity in the second wave. The prognosis was further deteriorated by comorbidities, highlighting the importance of early detection, efficient management, and adherence to safety procedures. Improving outcomes in future health emergencies requires bolstering maternal healthcare services and offering psychological support.

## Ethical clearance:

Ethical approval was obtained from the Ethics Committee of Gandhi Medical College, Bhopal.

## Sponsorship:

This study was not sponsored by any external agency.
